# Human‐host transcriptomic analysis reveals unique early innate immune responses in different sub‐phenotypes of COVID‐19

**DOI:** 10.1002/ctm2.856

**Published:** 2022-06-13

**Authors:** Ranjeet Maurya, Uzma Shamim, Partha Chattopadhyay, Priyanka Mehta, Pallavi Mishra, Priti Devi, Aparna Swaminathan, Sheeba Saifi, Kriti Khare, Aanchal Yadav, Shaista Parveen, Pooja Sharma, Vivekanand A, Akansha Tyagi, Vinita Jha, Bansidhar Tarai, Sujeet Jha, Mohd Faruq, Sandeep Budhiraja, Rajesh Pandey

**Affiliations:** ^1^ INtegrative GENomics of HOst‐PathogEn (INGEN‐HOPE) Laboratory CSIR‐Institute of Genomics and Integrative Biology (CSIR‐IGIB) Delhi India; ^2^ Academy of Scientific and Innovative Research (AcSIR) Ghaziabad India; ^3^ Max Super Speciality Hospital (A Unit of Devki Devi Foundation) Max Healthcare Delhi India

Dear Editor,

The diverse clinical manifestations of COVID‐19 are substantially modulated by host immune responses, emphasising the need for investigating the initial *Transcriptional* landscape leading to disease severity.^1^ This is the first study wherein host responses between sub‐phenotypes—mild, moderate, severe and mortality—have been elucidated to identify disease regulatory genes.

A total of 125 hospital‐admitted patients were categorised into sub‐phenotypes based on outcome (recovered, mortality) and disease severity (mild, moderate, severe). Following the Indian Council of Medical Research (ICMR) guidelines, shortness of breath (SOB), SpO_2_ and respiratory support (RS) requirements were used for severity classification. The clinical data highlighted that RS required by the patients did not always coincide with SpO_2_ levels and SOB, reinforcing analysis for differential host response with a single feature of RS requirement. The study design, including sample‐wise segregation into multiple clinical subgroups, SpO_2_ and level of RS requirement, is depicted in Figure S1A,B. The significance analysis of clinical data for sub‐phenotypes is provided in Tables S1 and S2. Pearson correlation analysis was performed to identify significant associations among clinical parameters (Figure S1C), specifically as demonstrated in Figure S1D–H. The methodology and literature supporting data interpretation are given as Supporting Information.

Foremost, we wanted to identify a possible mechanism leading to recovery from COVID‐19. Transcriptome analysis between recovered and mortality patients revealed 104 significant differentially expressed genes (DEGs), of which 93 were upregulated and 11 were downregulated (Supporting Information S1; Figure 1A). Several genes related to mucosal immunity (*MUC1, MUC4, MUC20*, *MUC21*),^2^ cell adhesion and cell–cell junction formation (*TJP1, PARD3, ACTN4, ACTG1, EMP1, PPL*), and cytoskeleton formation for epithelial cells (*KRT4, KRT78, KRT19, KRT80*, *KRT16*)^3^ were significantly upregulated in the recovered patients. The observed upregulated genes involved in the maintenance of epithelial integrity and mucosal immunity in recovered patients probably indicate an active defence against SARS‐CoV‐2 infection (Figure 1B). Pathway and network analysis (Figure 1C,D) concurred with the above results reflecting the maintenance of cell junction adherens and organisation of the protective airway epithelium.

**FIGURE 1:**
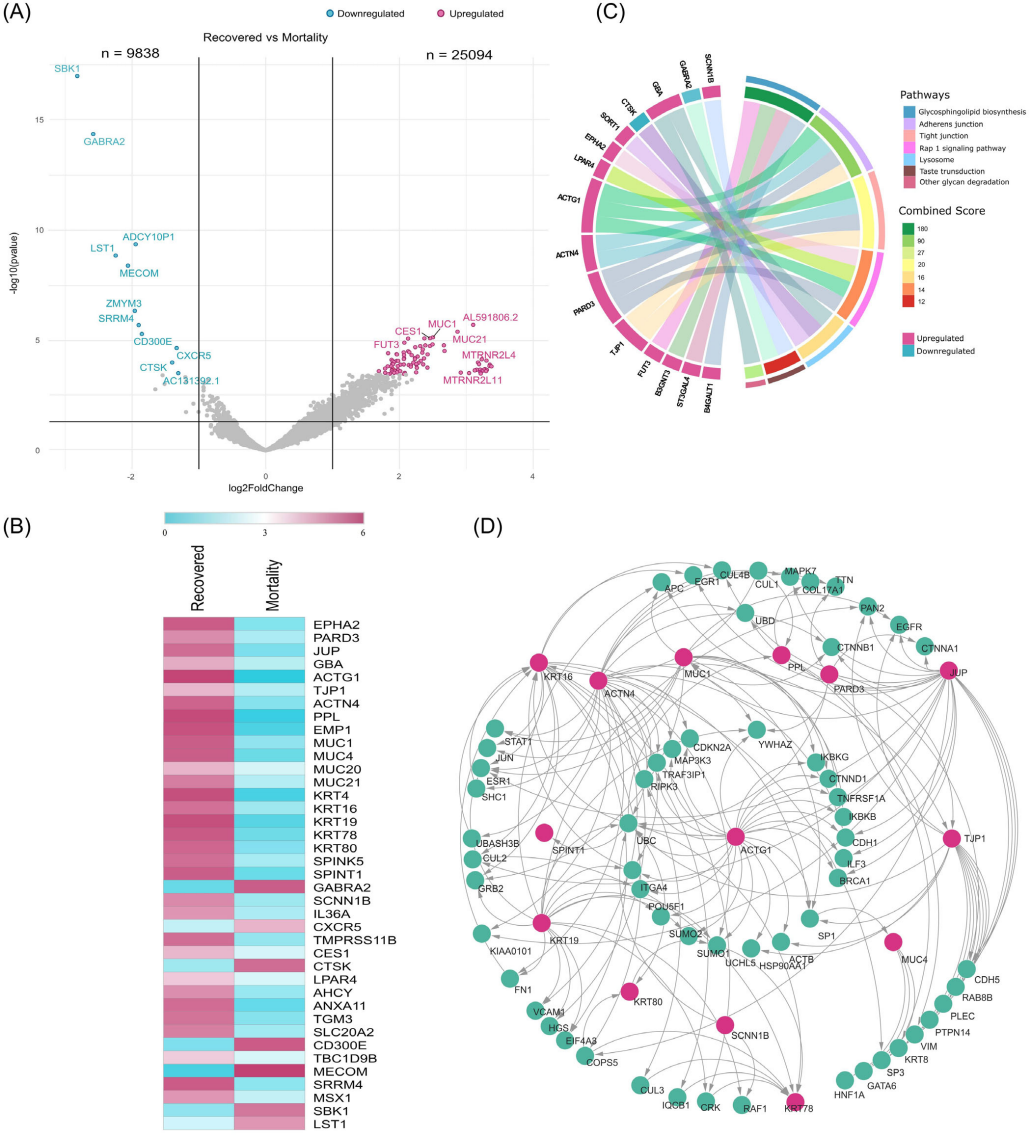
Differentially expressed genes (DEGs) in the recovered patients (compared to mortality) and functional analysis. (A) Volcano plot representing DEGs are shown highlighting genes with log2 fold change of ±1 and adjusted *p*‐value <.05. (B) The DEG profile of study‐selective genes on an average log2 scale of normalised counts per million. (C) Circos plot visualisation of enriched pathways obtained using Enrichr (KEGG database) with total significant DEGs; the combined score displays the significance of the gene set with their respective pathway. (D) Protein–protein interaction (PPI) network of study‐selective significant DEGs. The pink circles represent the upregulated genes, and the cyan circles denote interacting genes of the network

To understand differential disease severity within recovered, we looked further into the transcriptomic profile between mild, moderate, severe and mortality (Supporting Information S1; Figure 2A). Moderate and severe patients’ comparisons with mild revealed a distinct DEG profile, with 17 upregulated and two downregulated genes in moderate, whereas only six upregulated genes in severe compared to mild. Importantly, DEGs of the mortality patients highlighted the major downregulation of immune‐related genes: 30/43, 3/7, 21/24 versus mild/moderate/severe, respectively (Figure 2B). The association of significant DEGs with age using logistic regression analysis revealed the non‐dependence of the majority of genes (Supporting Information S2).

**FIGURE 2:**
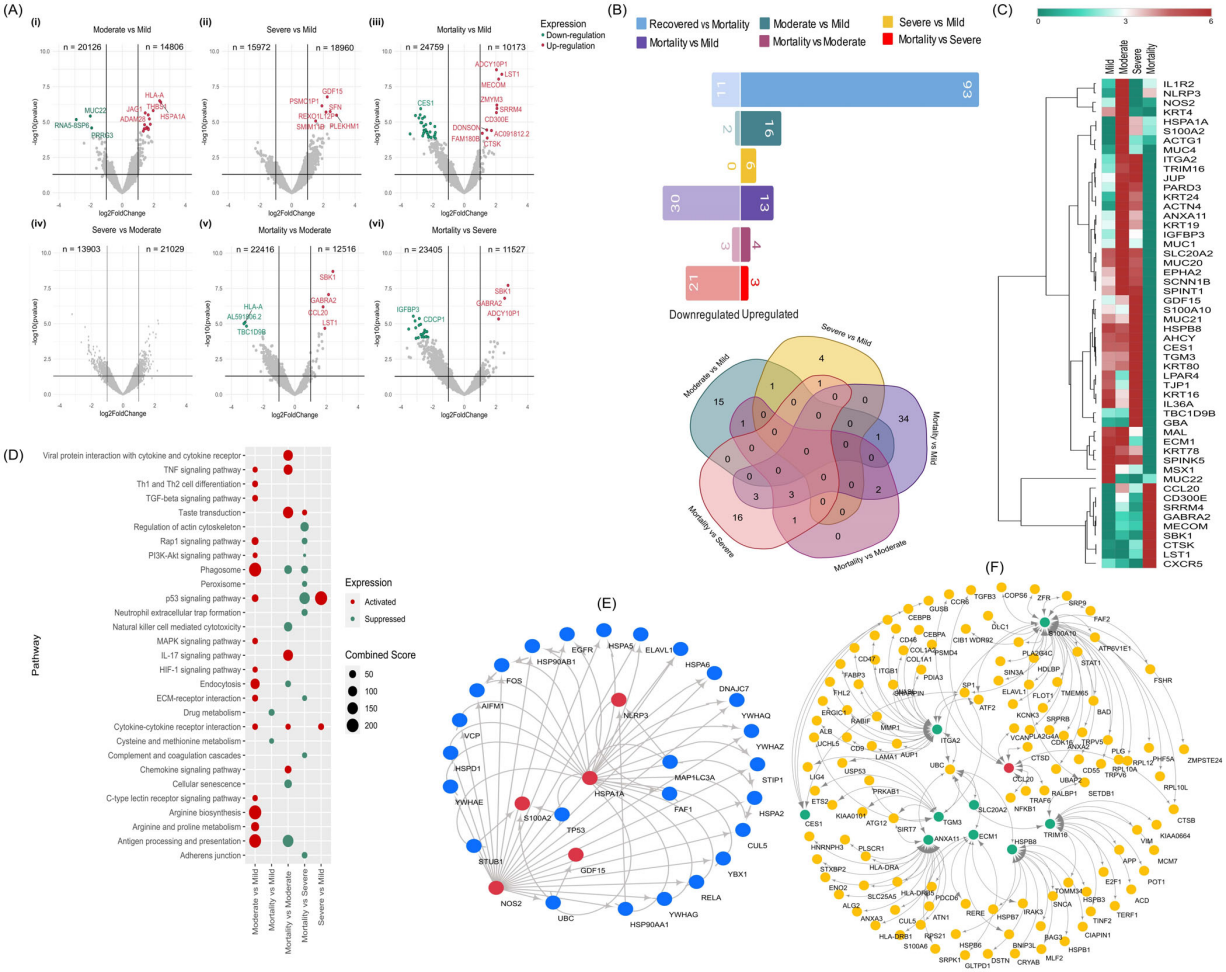
Differentially expressed genes (DEGs) of the severity sub‐phenotypes and functional analysis. (A) Volcano plot representing DEGs are shown with log2 fold change of ±1 and adjusted *p*‐value <.05 in different disease sub‐phenotypes; moderate versus mild, severe versus mild, mortality versus mild, severe versus moderate, mortality versus moderate, mortality versus severe. (B) Bar plot for the total count of significantly upregulated and downregulated genes in clinical sub‐phenotypes; Venn diagram illustrating the unique and shared significant DEGs among the comparison groups. (C) The DEG profile of study‐specific genes on an average log2 scale of normalised counts per million. (D) Dot plot visualisation of enriched pathways for different sub‐phenotypes; the colour of the dots represents upregulation and downregulation; the size of dots represents the combined score that displays the significance of the gene set with their respective pathway. (E and F) Protein–protein interaction (PPI) network of study‐specific DEGs across (E) moderate versus mild and (F) mortality versus mild, moderate and severe. The red circles represent the upregulated genes, the green circle represents the downregulated genes, and the small circles represent the interacting genes of the network

The upregulation of *IL1R2, HSPA1A, NLRP3, S100A2* and *NOS2* in moderate patients plausibly indicates a closely regulated antiviral innate immune response that provides protection from SARS‐CoV‐2 infection and prevents the hyperinflammatory response.^4^, ^5^, ^6^ The DEGs of the mortality group (versus mild/moderate/severe) were functionally different and showed overall decreased expression. Of significant interest was the downregulation of *MAL* (MYD88 adaptor‐like), an integral component of Toll‐like receptor (TLR) signalling during pathogen invasion,^7^ and *TRIM16*, which regulates inflammasome activity through NLRP1‐dependent production of IL‐1B (Interleukin) and IL‐18.^8^ *ECM1, HSPB8, TGM3, TMPRSS11B, ITGA2, SLC20A2, ANXA11, S100A10* and *IGFP3* were significantly downregulated in mortality patients, highlighting the possibility of a suboptimal innate immune response. The skewed upregulation of the chemokine *CCL20* in mortality patients can be an inflammatory effector molecule generated due to SARS‐CoV‐2 infection and might be integral to COVID‐19 severity.^9^ The heatmap highlights the differential abundance of genes across the clinical sub‐phenotypes (Figure 2C). Enrichment of pathways associated with antiviral inflammatory immune signalling (Figure 2D) and protein–protein interaction analysis highlighting the cellular stress response (Figure 2E,F) highlights the state of active defence in moderate patients and a suboptimal immune response in mortality.

Similarly, looking closely at the DEG profile between RS requirement subgroups: No‐RS, RS and ventilator support (VS), we identified distinct DEGs for VS compared to RS and No‐RS (Supporting Information S1; Figure 3A,B). Immune response genes with known roles in COVID‐19 and other infectious diseases, *IL22RA1, IFNE, CXCL14, CFD, CR2, IGHG3, IGLC2, C1QTNF4*, *C1QTNF7* and *PCDHA7*, were identified. The upregulation of several immune‐related genes within the VS patients suggests a hyperactivated host response, leading to enhanced levels of cytokines and interleukins (Figure 3C). Findings were corroborated by pathway (chemokine signalling, JAK–STAT signalling, alternate and classical pathway of the complement system) and network analysis (Figure 3D,E). The DEGs for RS versus No‐RS were similar to those of severity classification (Figure 3A–C).

**FIGURE 3:**
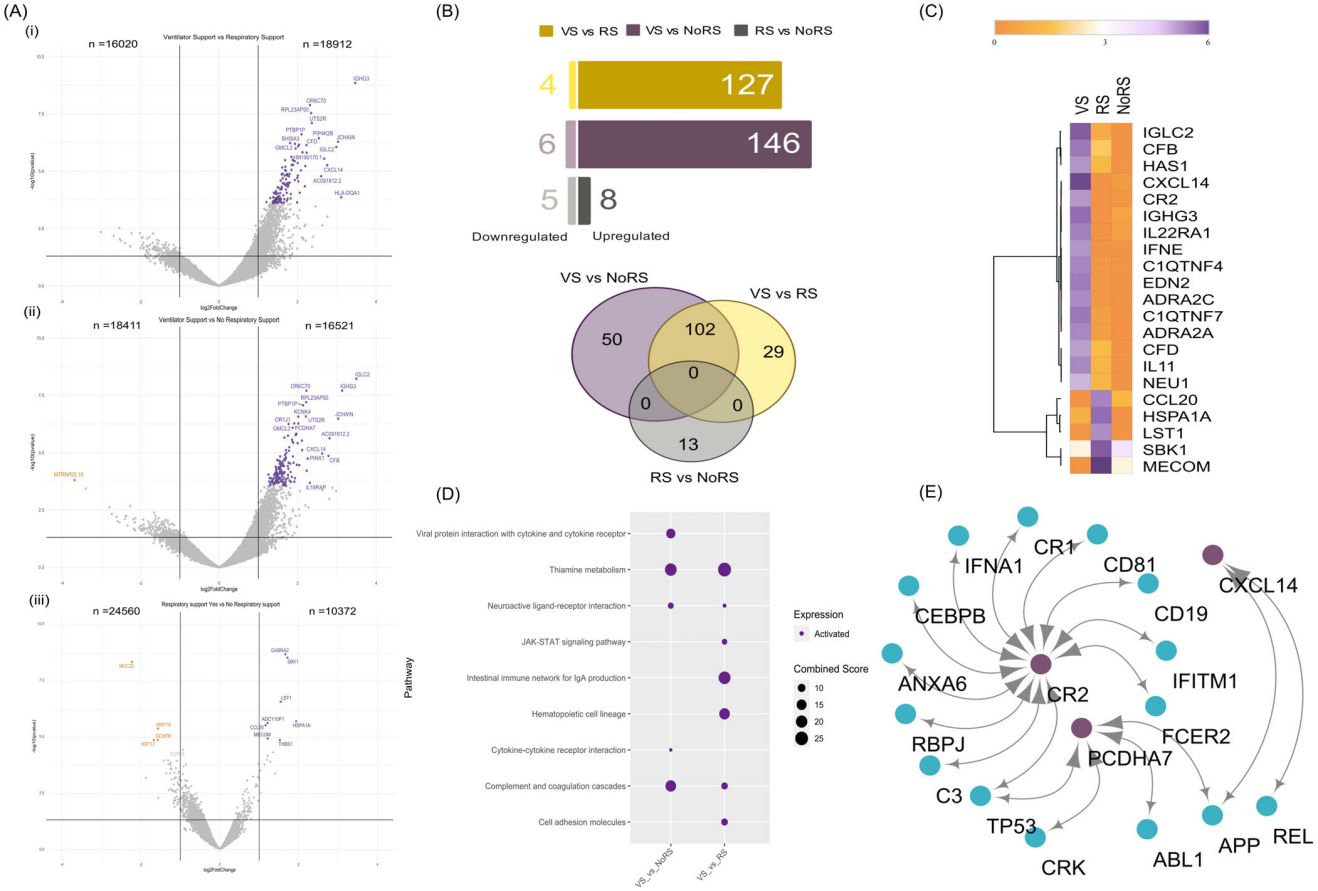
Differentially expressed genes (DEGs) of the ventilator support (VS)–respiratory support (RS)–No‐RS subgroups and functional analysis. (A) Volcano plots representing DEGs are shown with log2‐fold change of ±1 and adjusted *p*‐value <.05 in subgroups: (i) VS versus RS, (ii) VS versus No‐RS and (iii) RS versus No‐RS. (B) Bar plot for the significant count of upregulated and downregulated genes in clinical sub‐phenotypes; Venn diagram illustrating the unique and shared significant DEGs among the comparison groups. (C) The DEG profile of study‐specific genes on an average log2 scale of normalised counts per million. (D) Dot plot visualisation of enriched pathways for different sub‐phenotypes; the colour of the dots represents upregulation and downregulation; the size of dots represents the combined score that displays the significance of the gene set with their respective pathway. (E) Protein–protein interaction (PPI) network of study‐specific DEGs across VS versus RS/No‐RS. The violet circles represent the upregulated genes, and small circles represent the interacting genes of the network

Subsequently, we elucidated the possibly altered immune mechanisms in the clinical sub‐phenotypes. As illustrated in Figure 4A, upregulation of *S100A2* and *NLRP3* in moderate patients might provide a substantial immune‐inflammatory response. A possible counteractive effect is observed by the upregulation of *HSPA1A*, where *HSPA1A* leads to inhibition of‐Nuclear factor kappa B (NF‐κB)‐regulated NLR family pyrin domain containing 3 (NLRP3) inflammasome activation, thereby preventing exacerbation of inflammation.^10^ The mechanism for the deregulated host response due to downregulation of *MAL* and *TRIM16* in mortality patients is also depicted in Figure 4A. *MAL* facilitates recruitment of MyD88, affecting TLR‐2‐, TLR‐4‐ and RAGE‐mediated downstream signalling leading to activation of the NF‐κB pathway, the central regulator of innate immune signalling and inflammation. Figure 4B mechanistically illustrates the downstream signalling pathways affected by upregulated genes in VS patients. *IFNE*, a type I interferon, after sensing viral RNA is stimulated via Retinoic acid‐inducible gene 1 (RIG‐I) ‐like receptor signalling, establishing an antiviral response. *IFNE* and *IL22RA1* lead to subsequent activation of the Janus kinase (JAK)‐signal transducer and activator of transcription (STAT)  pathway, which, through *ISGF3* and *TFEB*, respectively, induces the expression of proinflammatory cytokines. Activation of the alternate complement pathway, directly by SARS‐CoV‐2 or via the JAK–STAT pathway, leads to endothelial and tissue injury. In summary, the expression of proinflammatory cytokines with tissue injury during the initial phase of SARS‐CoV‐2 infection can plausibly lead to a cytokine storm response in patients requiring VS. The study may benefit from longitudinal sampling of patients, different cohorts and variants of concern to understand the dynamic host response.

**FIGURE 4:**
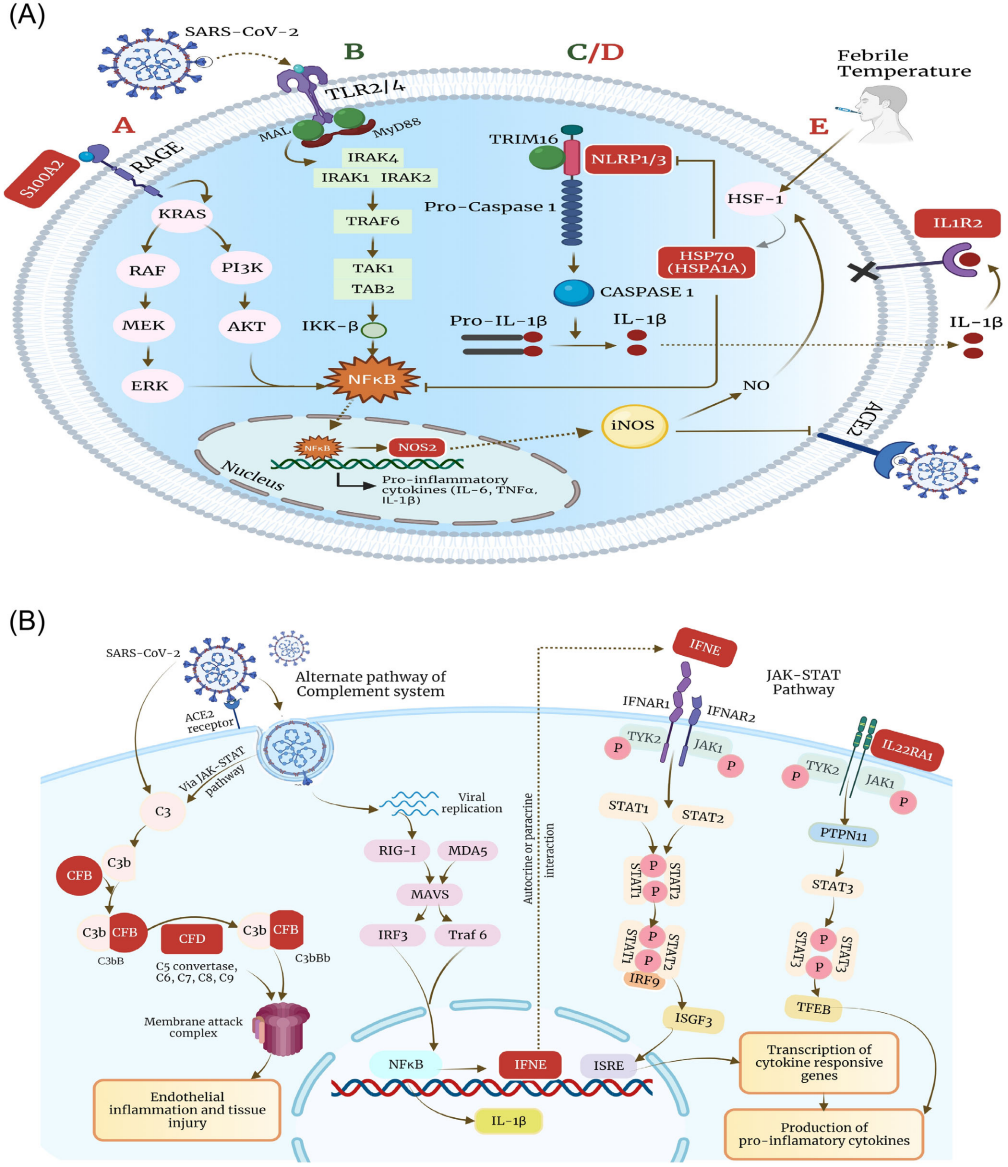
Schematic presentation of different immune signalling pathways. It highlights the role of deregulated genes in (A‐A) moderate; RAGE signalling pathway (A‐B) mortality; Toll‐like receptor (TLR) signalling (A‐C/D) mortality and moderate; NLRP1/3 inflammasome (A‐E) moderate; IL‐1 signalling pathway (B) ventilator support group; JAK/STAT signalling pathway; complement cascade activation. Upregulated genes are highlighted in red, and downregulated genes are highlighted in green

Our study provides evidence for the presence of distinct immune mechanisms due to SARS‐CoV‐2 infection modulating different COVID‐19 sub‐phenotypes. The initial host transcriptome profile may help understand the future disease severity and outcome in COVID‐19 patients. These findings can provide leads for prior targeted medical intervention and healthcare support.

## Manuscripts contribution to the field

Initial *Host Transcriptional* landscape of COVID‐19 sub‐phenotypes holds key to disease severity and clinical outcome.

Differential innate immune responses define moderate and mortality groups’ clinical phenotype.

Possible immune mechanisms identified in patient sub‐phenotypes can provide leads for targeted medical intervention and healthcare support.

## CONFLICT OF INTEREST

The authors declared that they have no conflict of interest exists.

## FUNDING INFORMATION

Bill and Melinda Gates Foundation (grant number: INV‐033578).

## Supporting information

Supporting information

Supporting information

Supporting information
