# Supplementary material for: Human‐host transcriptomic analysis reveals unique early innate immune responses in different sub‐phenotypes of COVID‐19
Source: Clin Transl Med. 2022 Jun 13;12(6):e856. doi: 10.1002/ctm2.856 (PMC9191825; doi:10.1002/ctm2.856)
Supplement: Supplementary file 1 — Supporting information [file CTM2-12-0-s003.docx]

**Title:** Human-host transcriptomic analysis reveals unique early innate immune responses in different sub-phenotypes of COVID-19

**Running title:** *Early immune responses in COVID-19 sub-phenotypes*

Ranjeet Maurya^1,2,#^, Uzma Shamim^1,#^, Partha Chattopadhyay^1,2,#^, Priyanka Mehta^1,$^, Pallavi Mishra^1,$^, Priti Devi^1,2,$^, Aparna Swaminathan^1^, Sheeba Saifi^1^, Kriti Khare^1,2^, Aanchal Yadav^1,2^, Shaista Parveen^1^, Pooja Sharma ^1,2^, Vivekanand A ^1,2^, Akansha Tyagi^3^, Vinita Jha^3^, Bansidhar Tarai^3^, Sujeet Jha^3^, Mohd Faruq ^1,2^, Sandeep Budhiraja^3^, Rajesh Pandey^1,2,*^

^1^INtegrative GENomics of HOst-PathogEn (INGEN-HOPE) laboratory, CSIR-Institute of Genomics and Integrative Biology (CSIR-IGIB), Mall Road, Delhi-110007, India.

^2^Academy of Scientific and Innovative Research (AcSIR), Ghaziabad-201002, India.

^3^Max Super Speciality Hospital (A Unit of Devki Devi Foundation), Max Healthcare, Delhi 110017, India.

^#^Joint First Authors

^$^Joint Second Authors

^*^Corresponding author

*** Contact Details:**

Rajesh Pandey, PhD

Principal Scientist,

INtegrative GENomics of HOst-PathogEn (INGEN-HOPE) laboratory,

CSIR-Institute of Genomics and Integrative Biology (CSIR-IGIB),

North Campus, Near Jubilee Hall, Mall Road, Delhi-110007, India.

Email: rajeshp@igib.in; Tel.: +91 9811029551

**Graphical Presentation:**

**
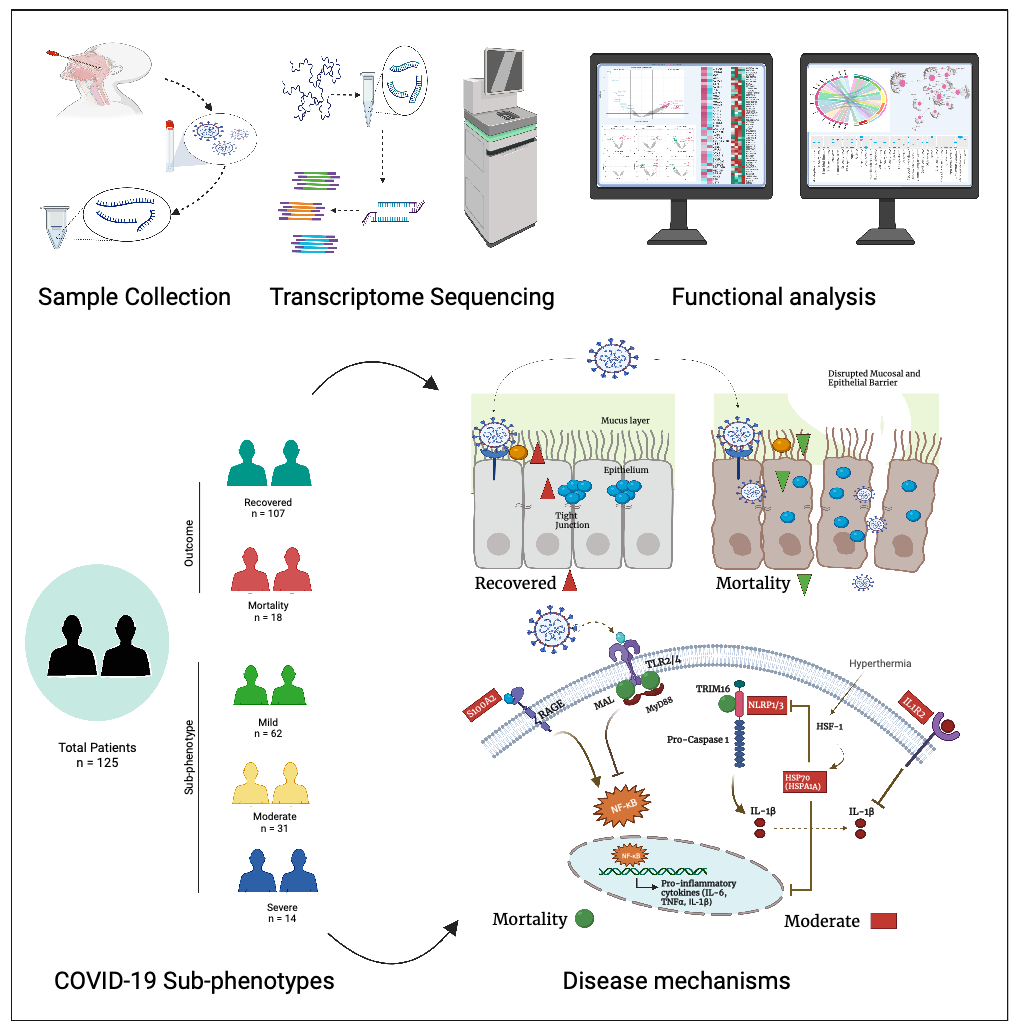
**

**Materials and Methods:**

**Sample Collection and Pre-processing**

The patients were admitted to the MAX Hospital, Delhi, India with confirmed COVID-19 positive status based on the RT-PCR results and followed by SARS-CoV-2 whole genome sequencing. The nasopharyngeal and/or throat swabs were collected in VTM solution by the paramedical staff at the hospital on the day of reporting, by trained medical staff with required safety precautions inclusive of PPE, face mask, and gloves.

**Clinical Subgrouping of Study Participants**

The patients were categorized into two broad groups based on the outcomes, viz., *Recovered* and *Mortality*. The recovered group was further subdivided into three sub-phenotypes based on disease severity - *Mild*, *Moderate* and *Severe*, as per Indian Council of Medical Research (ICMR) guidelines (Comprehensive Guidelines for Management of COVID-19 patients, Directorate General of Health Services, MoHFW, GOI). Briefly, SpO_2_ levels, requirement of respiratory support and/or breathlessness parameters were taken into consideration. In mild patients, the SpO_2_ level was ≥94% with no breathing problem. Moderate patients were defined as showing breathing difficulty with SpO_2_ levels ranging between 91-93%. Severe patients showed respiratory distress with respiratory support requirement and SpO_2_ levels <90%. Mortality group was defined as patients who succumbed to COVID-19 during hospital stay. Few samples which reported mild shortness of breath, despite having SpO_2_ >94%, were classified as *Mild* based on other symptoms presented by them. Similarly, patients who required respiratory support despite having SpO_2_ >94%, were classified as *Moderate* based on other symptoms presented by them.

Respiratory support is one of the distinct parameters for disease severity. We have also categorized the patients into three groups based on the requirement of respiratory support. Patients who did not require respiratory support were classified as *No RS*. The patients who required respiratory support were further categorized into two subgroups based on the type of respiratory support required. Patients, who required respiratory support, but not ventilator support, were classified as *RS*, and patients who required ventilator support were classified as *VS*.

It is important to mention that the clinical symptoms were recorded on the day of hospital admission for every patient when the patient reported positive for COVID-19 RT-PCR. Although, there can be differences in the time lapsed after the first day of symptom onset and reporting to hospital which is intrinsic to a disease in general and COVID-19 in particular.

**Viral RNA Isolation and qRT-PCR**

Viral RNA from VTM solutions was isolated using QIAmp viral mini kit, Qiagen, Cat. No. 52906 and SARS-CoV-2 detection and quantification was performed using TRUPCR SARS-CoV-2 kit (3B BlackBio Biotech India Ltd., Cat. No. 3B304) with a cycle threshold of 35.

**Library preparation and Sequencing**

A total of 250 ng of total RNA were taken for sequencing library preparation using Illumina TruSeq® Stranded Total RNA Library Prep Gold (cat. no 20020599) as per manufacturer’s reference guide (1000000040499 v00) and our previous study by Mehta et al. **^1^**. Briefly, cytoplasmic and mitochondrial rRNA were removed using biotinylated target specific oligos with Ribo-Zero rRNA removal beads. The purified RNA was fragmented using divalent cations under elevated temperature. The cDNA synthesis includes first strand cDNA prepared from the cleaved RNA fragments using reverse transcriptase and random primers, followed by second strand synthesis using DNA polymerase 1 and RNase H. The blunt 3’ end of the double stranded cDNA was adenylated, followed by addition of indexes and final amplification to enrich the library. The final library was purified using AMPure XP (Beckman Coulter, A63881). Agilent 2100 bioanalyzer was used to check the library quality, followed by denaturation using 0.2N NaOH and sequencing on NovaSeq 6000, using NovaSeq S2 v1.5 reagents at 2×101 read length and loading concentration of 400 pM.

**Quality control and read trimming**

The raw sequencing data was checked for quality using the FastQC program. The reads were then trimmed with Trimmomatic (v.0.39) **^2^** to remove low quality bases. The quality of reads was re-assessed with FastQC after this step to confirm quality improvements.

**Mapping to genome and Identification of differentially expressed genes (DEGs)**

The cleaned reads were then aligned to reference Human genome (assembly GRCh38.103) using Salmon (v1.4.0) **^3^**. Salmon provides fast and bias-aware quantification of transcript expression (Nature Methods). The reference genomes and reference annotation for Human were obtained from Ensemble database (http://ftp.ensembl.org/pub/release-103/gtf/homo_sapiens/). The sequence alignment files generated by Salmon v.1.1.0 (https://github.com/COMBINE-lab/salmon.git) were imported to R environment using tximport **^4^** for count-based differential gene identification using Bioconductor (https://www.bioconductor.org/packages/release/bioc/) package DESeq2 (v.1.30.1) **^5^**. The DESeq2 uses a generalised linear model for differential gene expression analysis and is based on negative binomial distribution. The normalisation was performed by calculating size factor using median-of-ratios method. Differential gene expression analysis was performed for the scenario status; recovered vs mortality, moderate/severe/mortality vs mild, severe/mortality vs moderate and mortality vs severe (severity classification). For respiratory support requirement groups, expression analysis was done between RS/VS vs No RS and RS vs No RS. The Benjamini-Hochberg correction was used to correct for multiple comparisons (with a FDR cut-off <0.05). Differentially expressed genes with adj p value < 0.05 and a log2 Fold Change of 1 were called as significant.

**KEGG Pathway analysis**

To identify the functionally enriched pathways differing between the comparison groups, Over Representation Analysis (ORA) was performed with significant differentially expressed genes (p-adj >0.05) using Enrichr against KEGG database **^6,7^.** Statistical significance of the pathways were calculated using Fisher’s Exact test. Pathways related with any infection and immune signalling, and with a *p value* cutoff of 0.1 were considered. The pathways were plotted using the ggplot2 R package, against the combined score and number of genes involved in the pathways.

**Network analysis and visualization**

Using Network Analyst tool, the protein-protein interaction (PPI) information were mapped to reveal the interactive links among DEGs (seed genes) and their interactive partners. The visualization of protein–protein interaction network map were constructed using standalone Cytoscape (v.3.8.2) software. Briefly, STRING interactome settings with a confidence score cut-off of 900 was used. The network was based upon protein-protein interaction (PPI).

**Statistical analysis**

Comparison between groups were described using descriptive statistics, which display continuous variables as medians or interquartile ranges and categorical variables as percentages or proportions. Wherever appropriate, we compared the differences using the Mann–Whitney *U* test, Kruskal Wallis test and Chi-square testing. Statistical significance of the pathways were calculated using Fisher’s Exact test.

**Data information supporting result interpretation:**

**Patient Cohort characterization: Classification and Clinical evaluation**

The present study was designed to co-analyse the host response as a function of phenotypic difference in severity of COVID-19 disease. All 125 patients were categorized into specific sub-phenotypes based on outcome, disease severity and level of respiratory support requirement. Based on disease outcome, 107 and 18 patients were grouped into Recovered and Mortality groups, respectively. Further sub-classification of recovered patients based on disease severity resulted in n=62 (mild), n=31 (moderate) and n=14 (severe). Also, based on the level of respiratory support (RS) requirement, 68 patients were grouped as No RS, 51 as RS and six patients in the ventilator support (VS) group; incidentally all six succumbed to SARS-CoV-2 infection. A total of 259 GB (4,846,696,474 bp) raw sequencing reads were generated from 125 COVID-19 transcriptomes, whereas, a median of ~17 million reads were generated for each sample. Downstream functional analysis includes pathway enrichment and Protein-Protein Interactions (PPI)-based network analysis. The sample-wise segregation of data into multiple groups, SpO_2_ level (peripheral oxygen saturation) and level of respiratory support requirement. Importantly, we observed that the level of respiratory support required did not always coincide with levels of SpO_2_ and shortness of breath (SOB) feature in the patients. This reinforced the need to analyse host response by using single feature of respiratory support requirement too. The patients’ clinical parameters evaluations are demonstrated in **Table S1**, wherein we noticed that specific clinical factors significantly correlate with differences in the disease severity and outcome. Pearson correlation analysis of the clinical parameters revealed a positive correlation between age and respiratory support **(Figure S1C)** which implied that older age group patients showed a higher propensity for respiratory support requirement. This is also reflected through **Figure S1D** where, the median age of the patients with mild COVID-19 symptoms was 51 years and was significantly different (*p-value < 0.001*) from moderate, severe and mortality patients (median age ~62) who required respiratory support. A similar trend was seen in the respiratory support-based classification, where median age of the No RS group (52.5 years) was significantly lower than the RS (61.5 years, p-value < 0.001) and VS group (59 years, p-value > 0.01) (**Table S2**). We also observed a negative correlation between the SpO_2_ levels and the requirement of respiratory support. The SpO_2_ levels were found to be significantly different when we compared severe group with either mild (*p-value* < 0.001) or moderate (p-value < 0.001) patients (**Figure S1E).** The same was true when we compared the SpO2 of No RS group with those who required respiratory support (*p-value* < 0.001) or ventilation support (*p-value* < 0.01) (**Figure S2**). However, no statistical significance was present in RS vs VS group. We also observed that the hospital stay days were higher for moderate (*p-value* < 0.001) and mortality (*p-value* < 0.01) groups as compared to the mild and also had a positive correlation with the requirement of respiratory support (**Figure S1F)**. The duration of hospital stay was also significantly different in No RS group compared to RS (*p-value* < 0.01) and VS group (*p-value* < 0.01). The percentage of patients reporting SOB was also significant in the No RS group when compared to RS (*p-value* < 0.01) and VS group (*p-value* > 0.01). It is important to highlight that the median Ct values for SARS-CoV-2 *E gene* (Ct 21.6) was lower (high viral load) in mortality cases when compared with the recovered group (Ct 25.7; (*p-value* > 0.01). Within the recovered group, the moderate group showed statistical difference for *RdRp gene* (*p-value* < 0.01) as well as for *E* (*p-value* < 0.01) in comparison to the mortality group (**Figure S1G & S1H).**

**Table S1: Demographic and clinical characteristics of COVID-19 patients at the time of hospital admission** (Patients’ segregation based on disease outcome and severity classification).

| Groups | Mortality | Recovered | | | | | |
| --- | --- | --- | --- | --- | --- | --- | --- |
|  | **(n=18)** | **(n=107)** | ***P*- value** | **Mild**  **(n=62)** | **Moderate**  **(n=31)** | **Severe**  **(n=14)** | ***P*-value** |
| Age | 61.5(53-68)* | 58(36-66) | 0.01^a^ | 51(25-68) | 62(53-69) | 61.5(52-68) | **0.001^b^** |
| Gender (F\|M) | 12\|5* | 66\|39* | 0.54^c^ | 36\|26 | 21\|10 | 10\|4 | 0.51^c^ |
| Ct value |  |  |  |  |  |  |  |
| *E* gene | 21.62(18.95-26.27)* | 25.7(21.16-28.69) | **0.03^a^** | 24.60(19.84-28.42) | 27.44(22.95-29.12) | 25.84(22.82-29.41%) | 0.18^b^ |
| *RdRp* Gene | 22.58(19.4-27.60)* | 26.42(21.06-29.29) | 0.13^a^ | 24.74(20.51-27.78) | 28.57(23.66-30.52) | 27.45(23.99-31.9) | **0.03^b^** |
| Hospital stays (days) | 16(7.5-21)* | 10(5-14)* | **0.05^a^** | 6.5(5-12) | 12(10-16) | 10(4.5-15)* | **0.002^b^** |
| SpO_2_ Level | 91(85-95)* | 97(94-98)* | **0.001^a^** | 97(96.5-98)* | 96(93-97)* | 80(75-85) | **0.001^b^** |
| Symptoms |  |  |  |  |  |  |  |
| Body ache | 5(27.8%) | 14(13.08) | 0.11^c^ | 10(16.12%) | 4(12.90%) | 0 |  |
| Sore throat | 6(33.33%) | 27(25.23%) | 0.47^c^ | 17(27.41%) | 7(22.58%) | 3(21.42%) | 0.83^c^ |
| Shortness Of Breath | 10(55.55%) | 42(39.25%) | 0.19^c^ | 19(30.64%) | 11(35.48%) | 10(71.42%) | **0.02^c^** |
| Cough | 8(44.44%) | 50(46.72%) | 0.86^c^ | 40(64.51%) | 12(38.7%) | 8(57.14%) | 0.06^c^ |
| Fever | 12(66.66%) | 81(75.70%) | 0.42^c^ | 48(77.41%) | 21(67.74%) | 12(85.71%) | 0.38^c^ |
| Comorbidities |  |  |  |  |  |  |  |
| Asthma/COPD | 0(0) | 6(5.60%) |  | 3(4.83%) | 1(3.22%) | 2(14.28%) | 0.30^c^ |
| Diabetes | 7(38.88%) | 33(30.84%) | 0.49^c^ | 16(25.80%) | 8(25.8%) | 9(64.28%) | **0.01^c^** |
| Heart disease | 3(16.66%) | 15(14.01%) | 0.77^c^ | 10(16.12%) | 3(9.67%) | 2(14.28%) | 0.69^c^ |
| Hypertension | 9(50%) | 41(38.31%) | 0.35^c^ | 22(35.48%) | 13(41.93%) | 6(42.85%) | 0.77^c^ |
| Thyroid | 2(11.11%) | 14(13.08%) | 0.82^c^ | 7(11.29%) | 3(9.67%) | 4(28.57%) | 0.18^c^ |
| Kidney disorders | 1(5.55%) | 9(8.41%) | 0.68^c^ | 2(3.22%) | 5(16.12%) | 2(14.28%) | 0.74^c^ |
| No Comorbidities | 6(33.33%) | 41(38.31%) | 0.69^c^ | 25(40.32%) | 11(35.48%) | 5(35.71%) | 0.88^c^ |

*Data are shown as median (IQR) or n(%);* *^a^Mann Whitney U test;* *^b^Kruskal Wallis test;* *^c^Chi2 test;* **missing data.*

*Values of significance are highlighted in bold.*

**Table S2: Demographic and Clinical characteristics of COVID-19 patients at the time of Hospital Admission (**Patients’ segregation based on level of respiratory support requirement).

|  | **No Respiratory Support** **(n=68)** | **Respiratory Support** **(n=51)** | **Ventilator Support** **(n=6)** | **p-value** |
| --- | --- | --- | --- | --- |
| **Age** | 52.5(28.5-64.5) | 61.5(53-69)* | 59(54-67) | **0.001^b^** |
| **Gender (M\|F)** | 41\|27 | 33\|16* | 6\|0 |  |
| **Ct value** |  |  |  |  |
| **E Gene** | 25.41(20.49-28.56) | 25.44(21.38-28.23)* | 23.77(15.72-27.9) | 0.7^b^ |
| **RdRp gene** | 25.27(20.74-28.52) | 27.21(21.57-29.77)* | 24.59(16.5-28) | 0.3^b^ |
| **Hospital stay (days)** | 8(5-12) | 12(7-17.5)* | 18(12-21) | **0.001^b^** |
| **SpO2 (%)** | 97(96-98)* | 91(85-96)* | 92(80.5-96)* | **0.001^b^** |
| **Symptoms** |  |  |  |  |
| Fever | 52(76.47%) | 38(74.50%) | 4(66.66%) | 0.86^c^ |
| Cough | 32(47.05%) | 23(45.09%) | 2(33.33%) | 0.81^c^ |
| Shortness of Breath | 21(30.88%) | 30(58.82%) | 3(50%) | **0.01^c^** |
| Body ache | 11(16.17%) | 6(11.76%) | 2(33.33%) | 0.37^c^ |
| Sore throat | 17(25%) | 12(23.52%) | 0 |  |
| Comorbidities |  |  |  |  |
| Diabetes mellitus | 20(29.41%) | 17(33.3%) | 4(66.6%) | 0.17^c^ |
| Hypertension | 26(38.23%) | 20(39.21%) | 2(33.33%) | 0.94^c^ |
| Cardiovascular Disease | 12(17.64%) | 7(13.72%) | 0 |  |
| Asthma\|COPD | 3(4.41%) | 2(3.92%) | 0 |  |
| Kidney disorder | 4(5.88%) | 7(13.72%) | 0 |  |
| Thyroid Disorder | 7(10.29%) | 8(15.62%) | 1(16.60%) | 0.63^c^ |
| **No Comorbidities** | 20(39.21%) | 26(38.23%) | 2(33.33%) | 0.92 ^c^ |

*Data are shown as median (IQR) or n(%);* *^a^Mann Whitney U test;* *^b^Kruskal Wallis test;* *^c^Chi2 test;* **missing data.*

*Values of significance are highlighted in bold.*

**Figure S1**


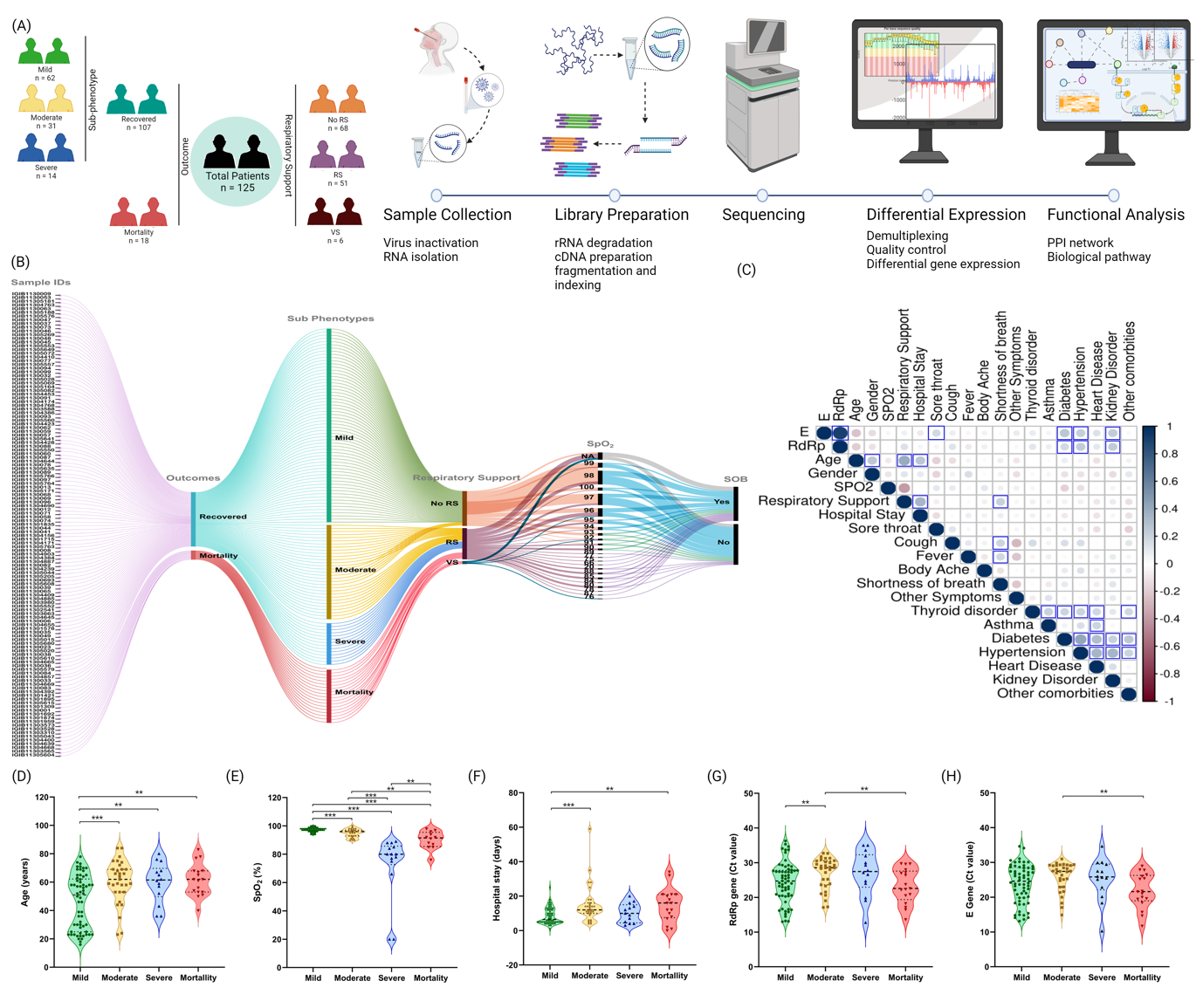


**Figure 1: Overview of study design and patient segregation with clinical characterization. (A)** Sample distribution and schematic workflow for Transcriptomic analysis followed by differentially expressed genes (DEG) analysis, downstream functional analysis and visualizations. **(B)** Sample wise segregation into multiple sub-phenotypes based on outcome, disease severity, level of respiratory support requirement, SpO2 and SOB. **(C)** Correlation matrix of clinical features for possible association between the variables; highlighting the significantly correlated variables in boxes. Significant variables like, **(D)** Age, **(E)** SpO_2_, **(F)** Hospital Stay, and **(G-H)** SARS-CoV-2 RT-PCR; Ct value for *RdRp* and *E* gene in four disease severity sub-phenotypes. The four groups represented as Mild (green), Moderate (yellow), Severe (blue) and Mortality (red), with statistical significance measured using Mann-Whitney *U* test [* represents p-value <0.05, ** represents p-value <0.01, *** represents p-value <0.001].

**Figure S2:**

**
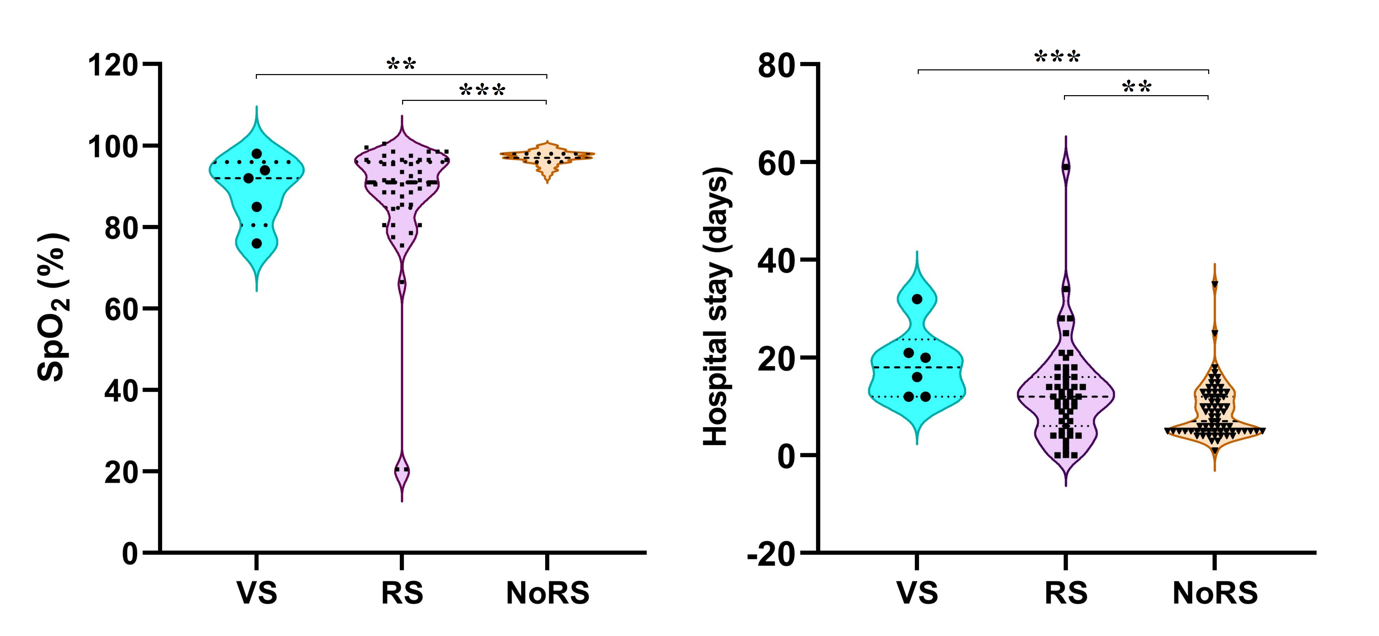
**

**Figure S2:** Significant variables like, SpO_2_ and Hospital Stay in the Respiratory group sub-phenotypes. The four groups represented as VS (cyan), RS (purple) and NoRS (brown), with statistical significance measured using Mann-Whitney *U* test [* represents p-value <0.05, ** represents p-value <0.01, *** represents p-value <0.001].

**Increased Epithelial Barrier Function and Mucosal Immunity behind Recovery from COVID-19**

To understand possible mechanism leading to recovery from COVID-19, we performed transcriptomic analysis between recovered and mortality patients. We observed that several genes related to mucosal immunity, cell adhesion and cell-cell junction were significantly upregulated in the recovered patients. A significant elevated expression of the various *MUC* genes, including *MUC1, MUC4, MUC21* (membrane-bound mucins), and *MUC21* (secreted mucin) was found in the recovered patients. These *MUC* genes produce the mucin protein that provides nasal mucosal defences by acting as a barrier against pathogens as well as contribute to innate immunity **^8^**. Emphasizing it further, *JUP* (junction plakoglobin), which is involved in the adherens junctions, desmosome structure formation and also an interactive protein of *MUC1*, also showed elevated expression in the recovered patients. Alongside, *TJP1, PARD3, ACTN4, ACTG1, EMP1* and *PPL* genes, involved in the formation of adherens and tight junctions were also upregulated in the recovered patients. The observed upregulation of genes involved in the maintenance of epithelial integrity and mucosal immunity in recovered patients, probably indicates an active defence against SARS-CoV-2 infection. This is also in conformity with the viral load difference between the two groups in our study, as well as a previous study**^9^**.

The Keratin family genes, *KRT4, KRT78, KRT19, KRT80* and *KRT16* of which *KRT4, KRT78* and *KRT19* have been previously reported in COVID positive patients **^10,11^** were also observed to have an elevated expression in the recovered patients. The keratin family proteins have a role in the formation of cytoskeleton of the epithelial cells which helps them maintain their cell integrity **^12^**. The serine proteinase inhibitors reduce the rate of SARS-CoV-2 infection by inhibiting membrane associated serine proteinase. Henceforth, the increased expression of serine proteinase inhibitor genes, *SPINK5* and *SPINT1,* in the recovered group might act as a protective shield compared to the mortality patients **^13^**.

We also found that the *GABRA2*, a negative regulator of taste sensation, is downregulated in the recovered patients whereas *SCNN1B*, involved in perception of salt taste through ENaC sodium channel, is upregulated in recovered group. Both the genes together indicate better taste perception in recovered group compared to those who succumbed to COVID-19.

Pathway analysis using KEGG database revealed adherens junction, tight junction, Rap1 signalling pathway (involved in increased endothelial barrier function), taste transduction and regulation of actin cytoskeleton pathways as major upregulated pathways in the recovered patients. Next, we built protein-protein interaction (PPI) networks to understand the interactions and functional associations of the study-specific genes obtained for this group **(Figure 2D)**. Together, the functional analysis reflects the concerted effort to maintain cell junction adherens and organization of the protective airway epithelial surfaces. However, neither the DEGs, nor the downstream functional analysis could highlight the differential immune/stress response between recovered and mortality groups, possibly due to diverse disease severity sub-phenotypes within the recovered patients. Thus, we investigated that aspect further.

**Different patterns of Immune Responses associated with COVID-19 sub-phenotypes**

To understand the possible mechanism behind differential disease severity trajectory within recovered patients, we looked further into the transcriptomic profile within disease sub-phenotypes. The comparison included six combinations; moderate, severe and mortality groups respectively with mild; severe and mortality groups respectively with moderate and mortality with severe. Overall, DEGs obtained for the mortality group (mortality vs mild; mortality vs severe; mortality vs moderate) were majorly downregulated when compared to other sub-groups (moderate vs mild; severe vs mild), indicating a negatively altered initial innate immune response especially in the mortality patients. Notably, no significantly DEGs were found in severe compared to moderate. Association of significant DEGs with Age, using logistic regression analysis revealed non-dependence of majority of genes **(SI-ii)**.

***Gene Expression in moderate vs mild & severe vs mild groups:*** The genes related to antiviral immune response, *IL1R2, HSPA1A, NLRP3* and *NOS2* were upregulated in the moderate patients compared to the mild. *HSPA1A*, a stress inducible gene, is known to be activated during viral infections due to febrile temperatures **^14^**. The upregulation of *HSPA1A* in our moderate conforms to the proposed mechanism of HSR (heat shock response) mediated upregulation of *HSPA1A* and subsequent regulation of infection-induced inflammatory response during COVID-19. *HSPA1A* is also known to repress the inflammation by suppressing NFkB and NLRP3 inflammasome, which has been shown to be upregulated in severe COVID-19 patients **^14,15^**. Further, *NOS2* is required for synthesis of NO which is an activator of COX-2 and inflammatory prostaglandins (PGs). The increased expression of *IL1R2*, decoy receptor for *IL1*, abolishes the aberrant IL1 signal transmission by competitively binding to IL1 **^16^**. Increased expression of *S100A2*, a S100 family calcium-binding proteins have crucial role in the regulation of immune homeostasis and inflammation, inducing the NFkB signalling through RAGE, TLR4 pathways and releasing proinflammatory cytokines at the site of infection **^17^**. Alongside, S100 family proteins are reported as prognostic markers for severe COVID-19 **^18^**. *GDF15*, another biomarker for COVID-19 severity, was also upregulated in our moderate patients. The fold change for *GDF15* was 1.4 in moderate (vs mild) which increased to 2.2 in severe (vs mild), indicative of its plausible association with disease severity.

***Gene Expression in mortality vs mild, mortality vs severe & mortality vs moderate:*** The DEGs of mortality group (vs mild/moderate/severe) were functionally different and showed an overall decreased expression when compared to other groups. Although the pathological significance of some DEGs in terms of COVID-19 is unknown, their significant association with mortality group might help understand prospective roles in COVID-19 disease severity. *MAL* gene, also known as MYD88 adaptor-like is an integral component of TOLL-like receptor signalling during pathogen invasion **^19^**. *HSPB8* assures proteolytic degradation of unfolded proteins, mediated by proteasomes or macroautophagy whereas *TBC1D9B*, a Ca^2+^-binding protein from TBC1 domain family, is a homolog of TBC1D9, which is recognized to phosphorylate and activate TBK1 for downstream autophagy processes during bacterial infections **^20^**.

*TRIM16* is also downregulated in mortality patients which regulates the activity of inflammasomes through NLRP1-dependent production of IL1B and IL18 **^21^**. Extracellular matrix protein 1 (*ECM1*) is a positive regulator for T_FH_ (T-follicular helper) differentiation and antibody response which is critical for humoral responses. *ECM1* deficiency is known to impair T(H)2 responses and reduced allergic airway inflammation in vivo **^22^**. Other genes, *TGM3, TMPRSS11B, ITGA2 SLC20A2, ANXA11, S100A10,* and *IGFP3* were significantly downregulated in the mortality groups, pointing towards possibility of sub-optimal innate immune response. Notably, *CES1*, is one of the key enzymes for converting the drug Remdesivir to its proactive form leading to its lung-specific accumulation and critical for the treatment of respiratory viral infection, such as COVID-19 **^23^**. The decreased expression of *CES1* gene might play a modulatory role during treatment regime provided to these patients. Interestingly, increased expression of *CCL20* (chemokine) and *CD300E* (activating receptor present on monocytes) in mortality group might be features leading to severe COVID-19 **^24,25^**.

***Pathway analysis for moderate vs mild, severe vs mild & mortality vs mild/moderate/severe*:** To understand the functional role of the genes, pathway analysis was done for all the DEGs across sub-phenotypes using KEGG database. Moderate patients (vs mild) showed enrichment of pathways for Rap1, MAPK, PI3K-Akt, p53 signalling pathways, Cytokine-cytokine receptor interaction, ECM-receptor interaction, Th1 and Th2 cell differentiation, TGF-beta signalling pathway, C-type lectin receptor signaling pathway, HIF1 signaling pathway, and TNF signaling pathway. Metabolic pathways such as arginine biosynthesis and arginine and proline metabolism were also enriched in moderate patients with impact on amino acid metabolism. Higher arginine biosynthesis and metabolism reflects increased NOS2 (Nitric oxide synthase), a pro-inflammatory cytokine being upregulated **^26,27^**. Three enriched pathways were present in the severe group - cytokine-cytokine receptor interaction and p53 signalling pathway. The mortality patients (when compared to mild, moderate and severe) highlighted pathways associated with antiviral immune responses such as IL17 signalling pathway, viral protein interaction with cytokine and cytokine receptor, TNF signalling pathway, Neutrophil extracellular trap formation and Natural killer cell mediated cytotoxicity pathways

***Network analysis for moderate vs mild, mortality vs mild/severe groups:*** PPI network of moderate patients (vs mild) identified *HSPA1A* and *NOS2* as major hub genes having a dense interacting network associated with cellular stress responses. The PPI network for mortality vs (mild, severe and mortality) presented TRIM16, ITGA2, TGM3, ANXA11, S100A10 and ITGA2 with highest interactions belonging to Cytokine Signaling in Immune system, Cellular responses to stress, antiviral immune responses, cell junction organization etc.

**Heightened initial immune and inflammatory response in Ventilated patients**

The need for respiratory support during COVID-19 disease is a critical factor which is correlated with the progression of the disease towards severity. It can also be considered as a surrogate marker for lung injury caused due to SARS-CoV-2 infiltration. Majority of the patients do not require respiratory support, while a subset transcends into a state where oxygen support is needed and a further subset requiring ventilator support. In order to understand the underlying transcriptomic differences in these patients, we conducted the functional analysis on DEGs obtained between the phenotypes. The comparison included three groups: respiratory support (RS) vs no respiratory support (No RS), ventilator support (VS) vs No RS and VS vs RS. We identified a total of 131 DEGs across VS vs RS and 152 DEGs across VS vs No RS, whereas the number of DEGs for RS vs No RS was 13.

A vast majority of genes (102) were found overlapping between VS vs RS and VS vs No RS groups, signifying a different gene expression profile associated with the ventilator group patients. Significant immune response related genes identified were *IL22RA1, IFNE, CXCL14, CFD, CR2, IGHG3, IGLC2, C1QTNF4, C1QTNF7* and PCDHA7. Many of these genes have been previously implicated with role in COVID-19 as well as other infectious diseases. *IFNE,* a type I interferon, after sensing viral RNA gets stimulated via RIG-I like receptor signalling and thus establishes antiviral response **^28^**. Cytokine *CXCL14*, mediates immune surveillance in epithelial cells along with promoting chemotactic activity for monocytes in lungs **^29^** and therefore can play a major role in immune cell infiltration during COVID-19 **^30^**. Interaction of IL22RA1 with IL22 and IL10 aids in downstream activation of JAK-STAT pathway, resulting in inflammation and tissue injury **^31^**. Complement factor D (CFD) and receptor CR2 are involved in initiating the alternate complement cascade **^32^**. *IGHG3* and *IGLC2* are reported to be associated with B-cell specific secreted immunoglobulin **^33^**.

Upregulation of *IL11* specifically in VS patients (vs RS) is known to trigger a heightened inflammatory response via JAK-STAT3 signalling pathway **^34^** and thus can be considered as potential initiators for cytokine storm. Another effector molecule, *HAS1* present in VS patients (vs No RS), acts as a pro-inflammatory cytokine because of its co-expression with *TGFB*, *IL1B*, and *TNFA* **^35^**. A significant upregulation of several immune-related genes within the VS patients, compared to the RS and No RS patients suggests a hyperactivated host immune response with aim to inhibit the proliferation of SARS-CoV-2 by releasing enhanced levels of cytokines and interleukins.

Several other genes that were upregulated in the VS patients, we did not find a direct association with immune response, yet their differential expression might play a role in disease severity. *NEU1* (neuraminidase 1) has been proposed to enhance lysosomal exocytosis, a mechanism that contributes to disease progression through release of SARS-CoV-2 virions into host cells **^36^**. Adrenoreceptors such as *ADRA2A* and *ADRA2C* have been reported for their non-specific binding to drugs resulting in respiratory difficulty and dry mouth condition; symptoms observed in our ventilator support patients **^37^**. *EDN2*, a potent vasoconstrictor, can lead to pulmonary hypertension **^38^** that can positively serve as a factor for enhancing disease severity **^39^**. The differentially expressed genes identified between RS and No RS groups were *CCL20*, *HSPA1A*, *MECOM, LST,* and *SBK1* similar to those obtained in severity classification. The pathways identified through KEGG include cytokine-cytokine receptor interaction, chemokine signalling, JAK-STAT signalling, complement and coagulation cascade and neuroactive ligand receptor interaction all of which are involved in eliciting a heightened inflammatory immune response. PPI network analysis of the overlapping study-selective genes for the ventilator support group identified *CR2, CXCL14* and *PCDHA7* with associated interactome.

**Putative Roles of differentially expressed genes in immune signalling pathways modulating distinct COVID-19 disease pathophysiology**

Subsequently, we elucidated the immune mechanisms that could be altered due to the deregulated genes in clinical sub-phenotypes of the patient cohort. During SARS-CoV-2 infection, the transcription of several pro-inflammatory cytokines including *IL6*, *TNFA*, *IL1B*, mostly relies on NFkB activation through the binding of ligands onto the receptors, present on the plasma membrane. Increased expression of *S100A2* (damage-associated molecular patterns) causes upregulation of NFkB pathway via activation of multi-ligand receptor, RAGE pathway contributing to inflammation. NFkB also mediates *NOS2* gene transcription which encodes inducible nitric oxide synthase (iNOS) enzyme that negatively regulates *ACE2* expression, thus preventing the viral entry inside the host cell. The other pathway found upregulated is the NLRP3 inflammasome formation, which mediates the synthesis of IL1B from pro-IL1B through caspase 1, inducing inflammation. Contrary to the immune activation observed through these pathways, febrile temperatures as well as NO, elicits *HSPA1A* expression, which can inhibit the NFkB complex formation and also negatively regulate NLRP3 inflammasome formation through HSR mechanism. Further, the secreted IL1B is sequestered and inactivated by its decoy receptor, IL1R2 at the cell surface resulting in reduced inflammatory response. Together, while the genes *S100A2, NLRP3* positively regulate the innate immune response in the moderate patients, *HSPA1A, NOS2* and *IL1R2* were observed to suppress the immune response, thereby indicating a balanced innate immune state in the moderate group patients. The mortality phenotype showed downregulation of the genes, *MAL* and *TRIM16*, which is known to affect the downstream activation of NFkB via Toll-like receptor (TLR) and NLRP3/1 receptor signalling, respectively. TLR 2/4 recognize the spike glycoprotein of SARS-CoV-2 upon infection, and via recruitment of MyD88 Adaptor Like Protein (*MAL*) culminate in the activation of NFkB, leading to downstream stimulation of innate immune response genes. However, the mortality patients in our cohort showed reduced production of pro-inflammatory cytokines due to impaired NFkB activation because of the downregulation of *MAL*. TRIM16, a member of TRIM family, increases IL1B production by binding to NLRP1 and Pro-caspase 1, thus enhancing the inflammatory response. In conclusion, the mortality group displayed an impaired immune response to SARS-CoV-2 infection.

The downstream signalling pathways affected due to upregulated genes in ventilator support patients is demonstrated as follows: Following SARS-CoV-2 interaction with ACE2 receptor at the host cell surface, RIG-I like receptors induce the expression of type-I interferon (IFNE). IFNE leads to subsequent activation of JAK-STAT pathway through its interaction with STAT1 and STAT2. Dimerization of STAT1 and STAT2 along with IRF9 leads to activation of ISRE (IFN stimulated response elements) that promotes the expression of pro-inflammatory cytokines. The receptor IL22RA1, also modulates the same pathway by activating TFEB and therefore induces the expression of pro-inflammatory genes. Furthermore, the JAK-STAT pathway is involved in lymphocyte differentiation, T helper cell regulation and assists in maintaining an antiviral state. Alternate complement pathway, directly activated by SARS-CoV-2 spike protein as well as via JAK-STAT pathway results in the formation of membrane attack complex that leads to endothelial and tissue injury.

**Detailed description of inferences pertaining to the study**

Transcriptomic analysis of patients’ sub-phenotypes at early infection stage during COVID-19 revealed prominent differences in the expression of immune related genes, a probable resultant of SARS-CoV-2 infection. Our results have been able to highlight underlying immune mechanisms which broadly correlate with the observed clinical sub-phenotypes. Recently, several studies have utilized nasal derived gene expression data sets to identify distinct host response in COVID-19 patients with different disease severity **^40-41^**, albeit while comparing with healthy controls. Does each clinical sub-phenotypes of COVID-19 possess initial transcriptomic features which can help understand the host response better and stratify the patients for priority medical interventions?

We found that clinical sub-phenotypes carried a distinct expression profile which could be associated with the pathological response. Our analysis with recovered patients versus mortality highlighted a heightened airway epithelial barrier function with restrictive tight junctions in the nasopharyngeal epithelium. . The dominance of Rap1 signaling pathway, along with the Regulation of actin cytoskeleton in the recovered patients, indicates the regulation of endothelial barrier since Rap1 induces signaling cascades for increased endothelial barrier function, and is important for the structural integrity of epithelial cells **^42,43^**. Evidence from several studies have indicated that SARS-CoV-2 targets the endothelium and causes vascular damage and dysfunction, leading to worsening in disease severity **^44,45^**. Furthermore, emerging data indicate that patients with COVID-19 infection exhibit hypertension, kidney disease, neurologic disorders, diabetes mellitus which are caused by severe endothelial cell dysfunction, accounting for a high mortality of COVID-19 patients **^46,47^**. A study has reported the downregulation of genes predominantly involved in cell adhesion activities such as cell–substrate adhesion, adherens junction organization, and focal adhesion assembly in BALF of deceased patients **^48^**. The existence of a corroborated effort to elevate the cell junction organization and epithelial integrity can be considered as a mechanism that reduces the permeability of the tissue for viral entry, thus controlling the infiltration of SARS-CoV-2 to lower respiratory airways **^49^**. Moreover, the upregulation of mucin genes especially *MUC1* is known to provide an active defence to viral infections **^50^**. *MUC1* expression at cell surface columnar epithelium has been shown to decrease the ability of the Influenza A virus to infect host cells, both in vitro and in vivo conditions **^51^**. In case of the respiratory syncytial virus, the *MUC1* gene plays an anti-inflammatory role by decreasing the TNF alpha release **^52^**. In other COVID-19 studies, the expression of *MUC1* and *MUC4* genes have been found to be upregulated **^53,54^**, where the mucosal immune system potentially inhibits the proliferation of SARS-CoV-2 at the primary site of viral infection (the nasopharynx).

Moderate patients showed balanced immune response, where activation of *NLRP3* gene may drive immune-inflammatory response towards severity. A counteractive effect could be provided by the upregulation of *HSPA1A*. The mechanism whereby *HSPA1A* leads to the inhibition of NF-κB regulated NLRP3 inflammasome activation thereby suppressing the pro-inflammatory cytokine release is well documented **^55,56^**. HSP70 proteins are shown to mitigate viral infections by controlling the innate and adaptive immune system as well as regulatory processes that dissemble inflammation, leading to prevention of cytokine storm that causes irreparable damage to organ tissues **^57^**. The resolution of COVID-19 disease in moderate patients can be modulated by the anti-inflammatory response of *HSPA1A* which can be considered for treatment modules. Importantly, in COVID-19, a strong inflammatory response is required, yet, can the physiological role of HSP70 be exploited for preventing exacerbation of inflammation leading to cytokine storm in patients prone to severe disease.

Another important effector molecule found in severe patients was *GDF15,* an anti-inflammatory cytokine, growth and differentiation factor whose expression is highly upregulated during pathological states involving inflammation, oxidative stress and hypoxia **^58^**. GDF15, a member of TGF-β superfamily, exerts its anti-inflammatory effects by directly regulating immune cells independent of centrally regulated mechanisms **^59^**.

Its levels are found increased with COVID-19 disease severity and acts as an inflammation-induced mediator of tissue tolerance **^60,61^**, an adaptation to restore a disturbed balance between pro- and anti-inflammatory cytokines. Precisely, a significant negative correlation of GDF15 with low SpO_2_ levels and poor respiratory function has been shown in COVID-19 patients as an indicator for Acute respiratory distress syndrome (ARDS) development **^62^**. Henceforth, the severe category patients might possess a high probability of developing ARDS requiring a vigilant early management. An intriguing finding of our study includes a subdued immune response in mortality patients as evident by the overall down regulation of immune related genes.

Decreased *MAL* expression may be a mediator of the observed suppressed innate immune response in the mortality patients. MYD88 adaptor-like protein (MAL) has been reported as a molecule of interest with diverse functions in generating appropriate immune responses. MAL gene facilitates the recruitment of MyD88, affecting TLR-2, TLR-4 and RAGE mediated downstream signalling leading to the activation of the NF-κB pathway, the central regulator of innate immune signalling and inflammation **^63^**. Independent of MYD88 adaptor function, MAL is also known to activate CREB via p38-MAPK and Mitogen-activated protein kinase kinase (MKK) signalling pathways **^64^**. In context of generating an early strong interferon response to curb infections, it has been reported that MAL is required for the production of type I interferon (IFN) downstream of TLR-9 stimulation with *Herpes simplex* virus **^65,66^**. Moreover, absence of MAL induced signaling has led to the exacerbated dissemination of *Bordetella pertussis* infection to the lungs leading to death in the mouse models **^67^**. Studies showed that a delayed Type I IFN response in BALB/c mice infected with SARS-CoV-2 led to enhanced viral replication resulting in elevated lung cytokine/chemokine levels, vascular leakage, and impaired T cell responses **^68^**. Taken together, a deficit in a strong interferon or innate immune response can cause SARS-CoV-2 infection to persist and cause damage that can lead to the activation of inflammatory molecules. In this context, the skewed activation of the chemokine, CCL20, in the mortality patients can be considered as inflammatory effector molecule generated due to SARS-CoV-2 infection as the airway epithelia inducibly expresses CCL20 during microbial infections **^69^**. CCL20 chemotaxis commensurate a signature of neutrophil and macrophage recruitment and activation **^70^**. Recently, two independent studies have proposed the increased production of CCL20 by SARS-CoV-2-infected inflammatory macrophages as causative factor for T cell recruitment and sustained alveolar inflammation, leading to critical illness in COVID-19 **^24,71^**. The current findings are summarized in **Figure S2.**

**
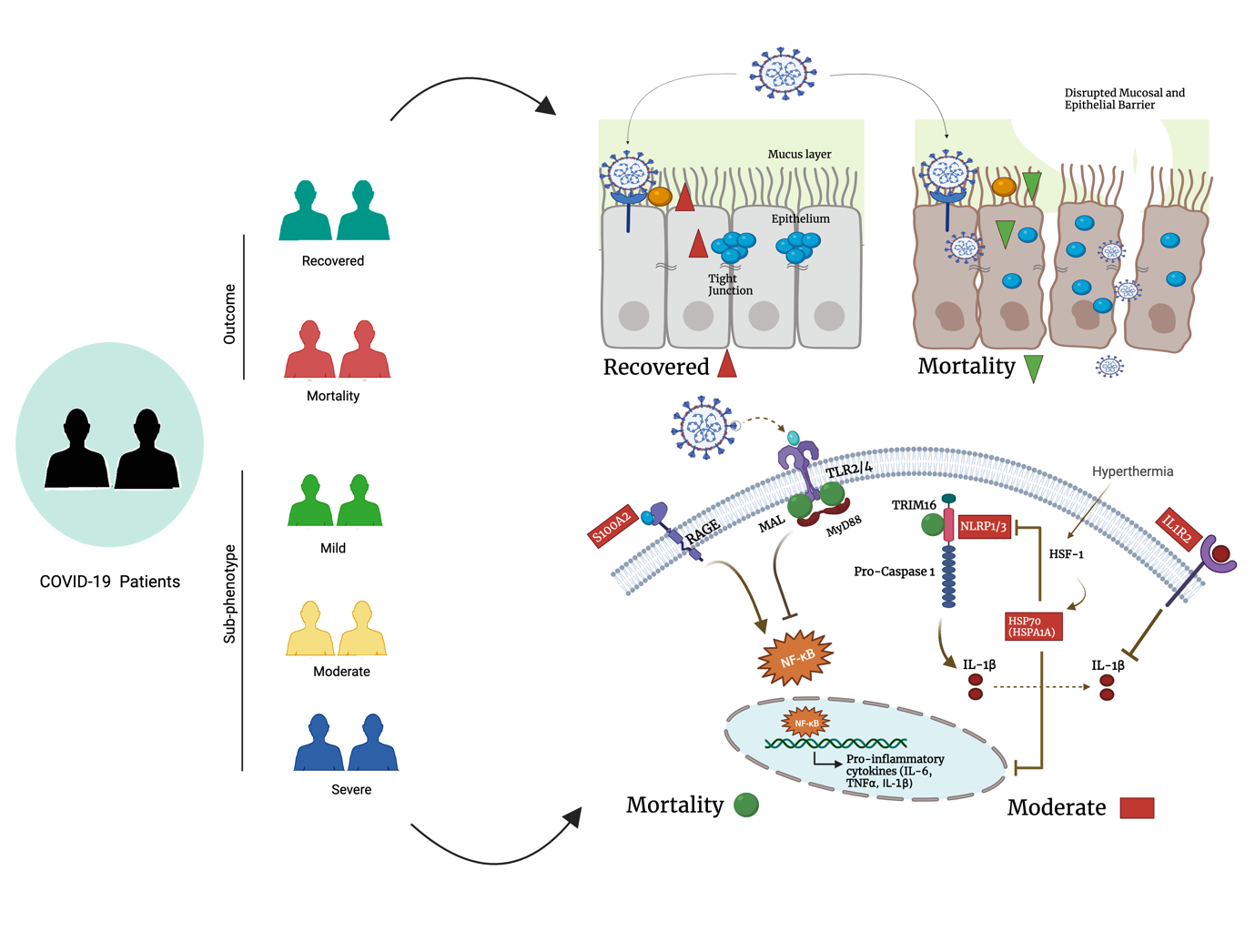
**

**Figure S2: Possible early host responses deciding COVID-19 disease trajectory:** Highlights epithelial barrier integrity and mucosal immunity for recovered patients; Differing immune mechanisms for moderate and mortality groups.

Surprisingly, a distinct gene expression profile was observed in patients who required mechanical ventilation in our cohort, although all these patients succumbed to COVID-19 and hence were part of the mortality patients. The presence of IFNE dominated Type 1 interferon response points toward a feeble antiviral response **^72^**, yet reported to play a role in mucosal immunity by elevating mucosal effector T-cell populations **^73^**. Just like the other Type I interferons, IFNE also exerts its biological activity by stimulating immune mediators and activating the JAK-STAT signalling pathways **^74^**. A recent study has proposed that Interferon (IFN) signalling via the JAK-STAT1 pathway is primarily responsible for alternate complement activation which can derail to sustainable hyperactivated state to produce deleterious effects of ARDS and lung injury in severe COVID-19 cases **^75^**. Since the gene signature observed in the ventilator patients was inflammatory, this mechanism might lead to the observed outcome.

Finally, several genes were observed with significant differential expression across multiple groups whose clinical relevance could not be ascertained through literature. Several classes of *MTRNR2L* (Humanin peptides), *SBK1*, *LST1*, *MECOM*, *MSX1* and *SRRM4* belonged to this category. These genes need further exploration, elucidation and understanding for its putative role in COVID-19 disease severity and modulating clinical outcome.

**References:**

1. Mehta P, Sahni S, Siddiqui S, et al. Respiratory Co-Infections: Modulators of SARS-CoV-2 Patients' Clinical Sub-Phenotype. *Front Microbiol*. 2021;12:653399.
2. Bolger AM, Lohse M, Usadel B. Trimmomatic: a flexible trimmer for Illumina sequence data. *Bioinformatics*. 2014;30(15):2114-2120.
3. Patro R, Duggal G, Love MI, Irizarry RA, Kingsford C. Salmon provides fast and bias-aware quantification of transcript expression. *Nat Methods*. 2017;14(4):417-419.
4. Soneson C, Love MI, Robinson MD. Differential analyses for RNA-seq: transcript-level estimates improve gene-level inferences. *F1000Res*. 2015;4:1521.
5. Love MI, Huber W, Anders S. Moderated estimation of fold change and dispersion for RNA-seq data with DESeq2. *Genome Biol*. 2014;15(12):550.
6. Chen EY, Tan CM, Kou Y, et al. Enrichr: interactive and collaborative HTML5 gene list enrichment analysis tool. *BMC Bioinformatics*. 2013;14:128.
7. Kanehisa M, Goto S. KEGG: Kyoto encyclopedia of genes and genomes. *Nucleic Acids Res*., 2000;28(1):27–30.
8. Rose MC, Voynow JA. Respiratory tract mucin genes and mucin glycoproteins in health and disease. *Physiol Rev*. 2006;86(1):245-278.
9. Robinot R, Hubert M, de Melo GD, et al. SARS-CoV-2 infection induces the dedifferentiation of multiciliated cells and impairs mucociliary clearance. *Nat Commun*. 2021;12(1):4354.
10. Mastropasqua L, Toto L, Chiricosta L, et al. Transcriptomic analysis revealed increased expression of genes involved in keratinization in the tears of COVID-19 patients. *Sci Rep*. 2021;11(1):19817.
11. Gisby J, Clarke CL, Medjeral-Thomas N, et al. Longitudinal proteomic profiling of dialysis patients with COVID-19 reveals markers of severity and predictors of death. *Elife*. 2021;10:e64827.
12. Ramms L, Fabris G, Windoffer R, et al. Keratins as the main component for the mechanical integrity of keratinocytes. *Proc Natl Acad Sci U S A*. 2013;110(46):18513-18518.
13. Fuentes-Prior P. Priming of SARS-CoV-2 S protein by several membrane-bound serine proteinases could explain enhanced viral infectivity and systemic COVID-19 infection. *J Biol Chem*. 2021;296:100135.
14. Guihur A, Rebeaud ME, Fauvet B, Tiwari S, Weiss YG, Goloubinoff P. Moderate Fever Cycles as a Potential Mechanism to Protect the Respiratory System in COVID-19 Patients. *Front Med (Lausanne)*. 2020;7:564170.
15. Heck TG, Ludwig MS, Frizzo MN, Rasia-Filho AA, Homem de Bittencourt PI. Suppressed anti-inflammatory heat shock response in high-risk COVID-19 patients: lessons from basic research (inclusive bats), light on conceivable therapies. *Clin Sci (Lond)*. 2020;134(15):1991-2017.
16. Peters VA, Joesting JJ, Freund GG. IL-1 receptor 2 (IL-1R2) and its role in immune regulation. *Brain Behav Immun*. 2013;32:1-8.
17. Sattar Z, Lora A, Jundi B, Railwah C, Geraghty P. The S100 Protein Family as Players and Therapeutic Targets in Pulmonary Diseases. *Pulm Med*. 2021;2021:5488591.
18. Biji A, Khatun O, Swaraj S, et al. Identification of COVID-19 prognostic markers and therapeutic targets through meta-analysis and validation of Omics data from nasopharyngeal samples. *EBioMedicine*. 2021;70:103525.
19. Fitzgerald KA, Palsson-McDermott EM, Bowie AG, et al. Mal (MyD88-adapter-like) is required for Toll-like receptor-4 signal transduction. *Nature*. 2001;413(6851):78-83.
20. Nozawa T, Sano S, Minowa-Nozawa A, et al. TBC1D9 regulates TBK1 activation through Ca^2+^ signaling in selective autophagy. *Nat Commun*. 2020;11(1):770.
21. Wei Y, Chen S, Wang M, Cheng A. Tripartite motif-containing proteins precisely and positively affect host antiviral immune response. *Scand J Immunol*. 2018;87(6):e12669.
22. Li Z, Zhang Y, Liu Z, et al. ECM1 controls T(H)2 cell egress from lymph nodes through re-expression of S1P(1). *Nat Immunol*. 2011;12(2):178-185.
23. Li R, Liclican A, Xu Y, et al. Key Metabolic Enzymes Involved in Remdesivir Activation in Human Lung Cells. *Antimicrob Agents Chemother*. 2021;65(9):e0060221.
24. Saris A, Reijnders TDY, Reijm M, et al. Enrichment of CCR6^+^ CD8^+^ T cells and CCL20 in the lungs of mechanically ventilated patients with COVID-19. *Eur J Immunol*. 2021;51(6):1535-1538.
25. Zenarruzabeitia O, Astarloa-Pando G, Terrén I, et al. T Cell Activation, Highly Armed Cytotoxic Cells and a Shift in Monocytes CD300 Receptors Expression Is Characteristic of Patients With Severe COVID-19. *Front Immunol*. 2021;12:655934.
26. Jia H, Liu C, Li D, et al. Metabolomic analyses reveals new stage-specific features of the COVID-19. *Eur Respir J*. 2021;2100284.
27. Xiao N, Nie M, Pang H, et al. Integrated cytokine and metabolite analysis reveals immunometabolic reprogramming in COVID-19 patients with therapeutic implications. *Nat Commun*. 2021;12(1):1618.
28. Rehwinkel J, Gack MU. RIG-I-like receptors: their regulation and roles in RNA sensing. *Nat Rev Immunol*. 2020;20(9):537-551.
29. Westrich JA, Vermeer DW, Colbert PL, Spanos WC, Pyeon D. The multifarious roles of the chemokine CXCL14 in cancer progression and immune responses. *Mol Carcinog*. 2020;59(7):794-806.
30. Jha PK, Vijay A, Halu A, Uchida S, Aikawa M. Gene Expression Profiling Reveals the Shared and Distinct Transcriptional Signatures in Human Lung Epithelial Cells Infected With SARS-CoV-2, MERS-CoV, or SARS-CoV: Potential Implications in Cardiovascular Complications of COVID-19. *Front Cardiovasc Med*. 2021;7:623012.
31. Gaudino SJ, Beaupre M, Lin X, et al. IL-22 receptor signaling in Paneth cells is critical for their maturation, microbiota colonization, Th17-related immune responses, and anti-Salmonella immunity. *Mucosal Immunol*. 2021;14(2):389-401.
32. Janeway CA Jr, Travers P, Walport M, et al. The complement system and innate immunity. New York: Garland Science; 2001. (pending)
33. Hsu HM, Chu CM, Chang YJ, et al. Six novel immunoglobulin genes as biomarkers for better prognosis in triple-negative breast cancer by gene co-expression network analysis. *Sci Rep*. 2019;9(1):4484.
34. Ng B, Cook SA, Schafer S. Interleukin-11 signaling underlies fibrosis, parenchymal dysfunction, and chronic inflammation of the airway. *Exp Mol Med*. 2020;52(12):1871-1878.
35. Siiskonen H, Oikari S, Pasonen-Seppänen S, Rilla K. Hyaluronan synthase 1: a mysterious enzyme with unexpected functions. *Front Immunol*. 2015;6:43.
36. Bongiovanni A, Cusimano A, Annunziata I, d'Azzo A. Sialylation of host proteins as targetable risk factor for COVID-19 susceptibility and spreading: A hypothesis. *FASEB Bioadv*. 2021;3(3):192-197.
37. Gordon DE, Jang GM, Bouhaddou M, et al. A SARS-CoV-2 protein interaction map reveals targets for drug repurposing. *Nature*. 2020;583(7816):459-468.
38. Stenmark KR, McMurtry IF. Vascular remodeling versus vasoconstriction in chronic hypoxic pulmonary hypertension: a time for reappraisal?. *Circ Res*. 2005;97(2):95-98.
39. Karmouty-Quintana H, Thandavarayan RA, Keller SP, Sahay S, Pandit LM, Akkanti B. Emerging Mechanisms of Pulmonary Vasoconstriction in SARS-CoV-2-Induced Acute Respiratory Distress Syndrome (ARDS) and Potential Therapeutic Targets. *Int J Mol Sci*. 2020;21(21):8081.
40. Ng DL, Granados AC, Santos YA, et al. A diagnostic host response biosignature for COVID-19 from RNA profiling of nasal swabs and blood. *Sci Adv*. 2021;7(6):eabe5984.
41. Jain R, Ramaswamy S, Harilal D, et al. Host transcriptomic profiling of COVID-19 patients with mild, moderate, and severe clinical outcomes. *Comput Struct Biotechnol J*. 2020;19:153-160.
42. Hanchard J, Capó-Vélez CM, Deusch K, Lidington D, Bolz SS. Stabilizing Cellular Barriers: Raising the Shields Against COVID-19. *Front Endocrinol*. 2020;11:583006.
43. Pannekoek WJ, Post A, Bos JL. Rap1 signaling in endothelial barrier control. *Cell Adh Migr*. 2014;8(2):100-107.
44. Kaur S, Tripathi DM, Yadav A. The Enigma of Endothelium in COVID-19. *Front Physiol*. 2020;11:989.
45. Quinaglia T, Shabani M, Breder I, Silber HA, Lima JAC, Sposito AC. Coronavirus disease-19: The multi-level, multi-faceted vasculopathy. *Atherosclerosis*. 2021;322:39-50.
46. Sardu C, Gambardella J, Morelli MB, Wang X, Marfella R, Santulli G. Hypertension, Thrombosis, Kidney Failure, and Diabetes: Is COVID-19 an Endothelial Disease? A Comprehensive Evaluation of Clinical and Basic Evidence. *J Clin Med*. 2020;9(5):1417
47. Cárdenas-Rodríguez N, Bandala C, Vanoye-Carlo A, et al. Use of Antioxidants for the Neuro-Therapeutic Management of COVID-19. *Antioxidants (Basel)*. 2021;10(6):971.
48. Li G, Wang J, He X, et al. An integrative analysis identifying transcriptional features and key genes involved in COVID-19. *Epigenomics*. 2020;12(22):1969-1981.
49. Linfield DT, Raduka A, Aghapour M, Rezaee F. Airway tight junctions as targets of viral infections. *Tissue Barriers*. 2021;9(2):1883965.
50. Chatterjee M, Huang LZX, Wang C et al. The glycosylated extracellular domain of MUC1 protects against SARS-CoV-2 infection at the respiratory surface [preprint]. https://doi.org/10.1101/2021.10.29.466408 Posted on bioRxiv October 29, 2021.
51. McAuley JL, Corcilius L, Tan HX, Payne RJ, McGuckin MA, Brown LE. The cell surface mucin MUC1 limits the severity of influenza A virus infection. *Mucosal Immunol*. 2017;10(6):1581-1593.
52. Li Y, Dinwiddie DL, Harrod KS, Jiang Y, Kim KC. Anti-inflammatory effect of MUC1 during respiratory syncytial virus infection of lung epithelial cells in vitro. *Am J Physiol Lung Cell Mol Physiol*. 2010;298(4):L558-L563.
53. Lu W, Liu X, Wang T, et al. Elevated MUC1 and MUC5AC mucin protein levels in airway mucus of critical ill COVID-19 patients. *J Med Virol*. 2021;93(2):582-584.
54. Plante JA, Plante KS, Gralinski LE, et al. Mucin 4 Protects Female Mice from Coronavirus. Pathogenesis [preprint]. http://dx.doi.org/10.1101/2020.02.19.957118 Posted on bioRxiv February 20, 2020.
55. Rébé C, Ghiringhelli F, Garrido C. Can the hyperthermia-mediated heat shock factor/heat shock protein 70 pathway dampen the cytokine storm during SARS-CoV-2 infection? *Br J Pharmacol*. 2020;10.1111/bph.15343.
56. Heck TG, Ludwig MS, Frizzo MN, Rasia-Filho AA, Homem de Bittencourt PI. Suppressed anti-inflammatory heat shock response in high-risk COVID-19 patients: lessons from basic research (inclusive bats), light on conceivable therapies. *Clin Sci (Lond)*. 2020;134(15):1991-2017.
57. Evans SS, Repasky EA, Fisher DT. Fever and the thermal regulation of immunity: the immune system feels the heat. *Nat Rev Immunol*. 2015;15(6):335-349.
58. Myhre PL, Prebensen C, Strand H, et al. Growth Differentiation Factor 15 Provides Prognostic Information Superior to Established Cardiovascular and Inflammatory Biomarkers in Unselected Patients Hospitalized With COVID-19. *Circulation*. 2020;142(22):2128-2137.
59. Rochette L, Zeller M, Cottin Y, Vergely C. GDF15: an emerging modulator of immunity and a strategy in COVID-19 in association with iron metabolism. *Trends Endocrinol Metab*. 2021;32(11):875-889.
60. Notz Q, Schmalzing M, Wedekink F, et al. Pro- and Anti-Inflammatory Responses in Severe COVID-19-Induced Acute Respiratory Distress Syndrome-An Observational Pilot Study. *Front Immunol*. 2020;11:581338.
61. Rochette L, Zeller M, Cottin Y, Vergely C. Insights Into Mechanisms of GDF15 and Receptor GFRAL: Therapeutic Targets. *Trends Endocrinol Metab*. 2020;31(12):939-951.
62. Alserawan L, Peñacoba P, Orozco Echevarría SE, et al. Growth Differentiation Factor 15 (GDF-15): A Novel Biomarker Associated with Poorer Respiratory Function in COVID-19. *Diagnostics (Basel)*. 2021;11(11):1998.
63. Battagello DS, Dragunas G, Klein MO, Ayub ALP, Velloso FJ, Correa RG. Unpuzzling COVID-19: tissue-related signaling pathways associated with SARS-CoV-2 infection and transmission. *Clin Sci (Lond)*. 2020;134(16):2137-2160.
64. Belhaouane I, Hoffmann E, Chamaillard M, Brodin P, Machelart A. Paradoxical Roles of the MAL/Tirap Adaptor in Pathologies. *Front Immunol*. 2020;11:569127.
65. Bonham KS, Orzalli MH, Hayashi K, et al. A promiscuous lipid-binding protein diversifies the subcellular sites of toll-like receptor signal transduction. *Cell*. 2014;156(4):705-716.
66. Zyzak J, Mitkiewicz M, Leszczyńska E, Reniewicz P, Moynagh PN, Siednienko J. HSV-1/TLR9-Mediated IFNβ and TNFα Induction Is Mal-Dependent in Macrophages. *J Innate Immun*. 2020;12(5):387-398.
67. Bernard NJ, Finlay CM, Tannahill GM, Cassidy JP, O'Neill LA, Mills KH. A critical role for the TLR signaling adapter Mal in alveolar macrophage-mediated protection against Bordetella pertussis. *Mucosal Immunol*. 2015;8(5):982-992.
68. Hadjadj J, Yatim N, Barnabei L, et al. Impaired type I interferon activity and inflammatory responses in severe COVID-19 patients. *Science*. 2020;369(6504):718-724.
69. Starner TD, Barker CK, Jia HP, Kang Y, McCray PB Jr. CCL20 is an inducible product of human airway epithelia with innate immune properties. *Am J Respir Cell Mol Biol*. 2003;29(5):627-633.
70. Li Q, Laumonnier Y, Syrovets T, Simmet T. Recruitment of CCR6-expressing Th17 cells by CCL20 secreted from plasmin-stimulated macrophages. *Acta Biochim Biophys Sin (Shanghai)*. 2013;45(7):593-600.
71. Saris A, Reijnders TDY, Reijm M, et al. Enrichment of CCR6+ CD8+ T cells and CCL20 in the lungs of mechanically ventilated patients with COVID-19. *Eur J Immunol*. 2021;51(6):1535-1538.
72. Day SL, Ramshaw IA, Ramsay AJ, Ranasinghe C. Differential effects of the type I interferons alpha4, beta, and epsilon on antiviral activity and vaccine efficacy. *J Immunol*. 2008;180(11):7158-7166.
73. Xi Y, Day SL, Jackson RJ, Ranasinghe C. Role of novel type I interferon epsilon in viral infection and mucosal immunity. *Mucosal Immunol*. 2012;5(6):610-622.
74. Zwarthoff EC, Mooren AT, Trapman J. Organization, structure and expression of murine interferon alpha genes. *Nucleic Acids Res*. 1985;13(3):791-804.
75. Yan B, Freiwald T, Chauss D, et al. SARS-CoV-2 drives JAK1/2-dependent local complement hyperactivation. *Sci Immunol*. 2021;6(58):eabg0833.
